# MiR-122-5p regulates erastin-induced ferroptosis via CS in nasopharyngeal carcinoma

**DOI:** 10.1038/s41598-024-59080-w

**Published:** 2024-05-01

**Authors:** Liqing Guo, Zhi Wang, Yanpeng Fu, Shuhong Wu, Yaqiong Zhu, Jiasheng Yuan, Yuehui Liu

**Affiliations:** Department of Otolaryngology, The Second Affiliated Hospital, Jiangxi Medical College, Nanchang University, Nanchang, People’s Republic of China

**Keywords:** Nasopharyngeal carcinoma, miR-122-5p, CS, Ferroptosis, Cancer, Oncology

## Abstract

Nasopharyngeal carcinoma (NPC) is a tumor that occurs in the nasopharynx. Although advances in detection and treatment have improved the prognosis of NPC the treatment of advanced NPC remains challenging. Here, we explored the effect of microRNA (miR)-122-5p on erastin-induced ferroptosis in NPC cells and the role of ferroptosis in the development of NPC. The effect of miR-122-5p silencing and overexpression and the effect of citrate synthase on erastin-induced lipid peroxidation in NPC cells was analyzed by measuring the amounts of malondialdehyde, Fe^2+^, glutathione, and reactive oxygen species and the morphological alterations of mitochondria. The malignant biological behavior of NPC cells was examined by cell counting kit-8, EDU, colony formation, Transwell, and wound healing assays. The effects of miR-122-5p on cell proliferation and migration associated with ferroptosis were examined in vivo in a mouse model of NPC generated by subcutaneous injection of NPC cells. We found that erastin induced ferroptosis in NPC cells. miR-122-5p overexpression inhibited CS, thereby promoting erastin-induced ferroptosis in NPC cells and decreasing NPC cell proliferation, migration, and invasion.

## Introduction

The nasopharyngeal epithelium is the site of the common head and neck cancer known as nasopharyngeal carcinoma (NPC). It has a distinctive geographic distribution and is most prevalent in East and Southeast Asia^1^. Approximately 129,000 patients with NPC were reported worldwide in 2018^2^. NPC has been linked to the Epstein-Barr (EB) virus, genetics, and environmental factors^3–5^. The anatomical location of the nasopharynx makes early detection of NPC difficult, and in its advanced stages, NPC is prone to recurrence and metastasis, resulting in inadequate therapeutic outcomes^4,6^. Despite recent advances in diagnosis and treatment, further investigation of the etiology of NPC and the identification of therapeutic targets are important issues.

Ferroptosis is a type of iron-dependent cell death associated with increased lipid peroxidation^7^. Acyl-CoA5 synthetase long-chain family member 4 (ACSL4) promotes ferroptosis by catalyzing the peroxidation of polyunsaturated fatty acids (PUFAs) and inducing the formation of reactive oxygen species (ROS)^8,9^. Glutathione peroxidase 4 (GPX4), which is part of the human antioxidant system, is a target for regulating ferroptosis^10,11^, and GPX4 knockdown promotes ferroptosis^12^. The occurrence of ferroptosis can be detected by changes in mitochondrial morphology observed under a transmission electron microscope, as well as by measuring the levels of Fe^2+^, ROS, and ROS-related products^7,13^. Among them, the morphological changes of cells (especially mitochondria) during ferroptosis are important features that distinguish ferroptosis from other forms of programmed cell death, such as apoptosis, autophagy and necrosis. When ferroptosis occurs, mitochondria often undergo changes such as membrane shrinkage, membrane density increase, membrane rupture, and ridge reduction^14,15^. Erastin is a ferroptosis-inducing compound that acts by inhibiting cystine-glutamate reverse transporters on cell membranes^16,17^. Ferrostatin-1 (Fer-1) is antioxidant that inhibits ferroptosis by inhibiting lipid peroxidation^18,19^. Ferroptosis affects the development of a variety of diseases, including tumors, autoimmune diseases, and neurodegenerative diseases^20–22^. Several compounds exert anticancer effects in NPC through ferroptosis^23–25^, and ferroptosis is related to radiation sensitivity in NPC^26^.

MicroRNAs (miRNAs) are non-coding RNA molecules approximately 21–25 nucleotides long that target the 3′-untranslated region of specific genes and regulate several metabolic pathways by modulating gene transcription and translation^27^. miRNAs can regulate tumor incidence and development by controlling tumor cell growth, invasion, migration, and chemotherapeutic resistance^28^. miR-122-5p is involved in regulating the development of several malignant tumors such as gastric cancer, renal cell carcinoma, liver cancer, melanoma, and breast cancer^29–33^. Work from our group demonstrated that miR-122-5p regulates NPC cell proliferation, migration, and invasion^34^. miRNAs modulate target genes in gastric cancer, liver cancer, melanoma, and other tumor cells to inhibit or promote ferroptosis^35–37^. However, whether miR-122-5p affects ferroptosis in NPC remains unknown.

Bioinformatics prediction identified citrate synthase (CS) as a target of miR-122-5p regulation. CS is the rate-limiting enzyme in the tricarboxylic acid cycle, which is linked to energy stress and oxidative phosphorylation^38^. The activity of CS is positively associated with the malignant biological behavior of several tumors, including hepatocellular carcinoma, ovarian cancer, and pancreatic cancer^39–41^. However, the effect of CS on NPC is not clear.

In this study, we investigated the association between miR-122-5p, ferroptosis, and CS. The results showed that miR-122-5p specifically targeted CS and promoted erastin-induced ferroptosis, which in turn decreased cell proliferation, migration, and invasion in NPC. In vivo experiments in nude mice verified that miR-122-5p inhibited the growth of NPC cells through ferroptosis. The present findings provide new insight into the mechanism underlying the effect of miR-122-5p on NPC.

## Materials and methods

### Patients

A total of 15 tumor samples were obtained from patients who visited the Second Affiliated Hospital of Nanchang University and were pathologically diagnosed with NPC but did not undergo radiation or chemotherapy. Patients who presented to the Second Affiliated Hospital of Nanchang University with a pathological diagnosis of nasopharyngeal lymphatic hyperplasia donated 15 normal tissue samples. The Ethics Committee of the Second Affiliated Hospital of Nanchang University approved the study.

### Cell culture

The nasopharyngeal mucosal cell line NP69 and the NPC cell lines CNE-1, CNE-2Z, 5-8F, and 6-10B were obtained from Cell Bank of the Chinese Academy of Sciences in Shanghai. All cells were cultured in DMEM (Solarbio, China) or 1640 (Solarbio, China) media with 10% FBS (TransGen, China) in a cell incubator with 5% CO_2_.

### Mimics and inhibitor cell transfection

Cells were inoculated at a density of 5 × 10^5^/well and transfected when reaching 50–70% confluency. According to the instructions, 250 μl of ribozyme free water was added to reagent tubes containing miR-122-5p mimics or miR-122-5p inhibitor (catalog numbers miR10000421 and miR20000421, RiboBio, China). For transfection, 10 μl R-fect (small nucleic acid transfection reagent) (Biodai, China) was mixed with 7.5 μl miR-122-5p mimics or inhibitor and incubated for 20 min miR-NC (with the sequence 5'-UUC UCC GAA CGU GUC ACG U-3') was also transfected as a negative control. The incubated transfection reagents were added to the cells and supplemented with serum-free medium to a volume of 2 ml. After 8 h, the complete medium was replaced and continued to culture for 48–72 h.

### Lentiviral transfection

Cells (1 × 10^5^) were uniformly inoculated in a 6-well cell culture plate. On the second day, the corresponding virus volume was added to the cells according to the MOI value, and the fresh culture medium was replaced after 24 h. The fluorescence was visualized using a fluorescence microscope the next day, and 1–2 μg/ml of purinomycin was added for screening when 90–100% of cells were fluorescent and in good condition. After continuous screening and three passages, the stable cell lines were selected for follow-up experiments.

### Quantitative real time-polymerase chain reaction (qRT-PCR)

The Trizol reagent (TransGen Biotech, China) was used for extracting total cell or tissue RNA according to the manufacturer’s instructions. The extracted RNA was then reverse transcribed into cDNA using a cDNA synthesis kit (TransGen Biotech, China). The SYBR Green Mix kit (TaKaRa, Japan) was used for qRT-PCR detection. U6 or GAPDH was used as the internal control, and the 2^−∆∆ct^ method was used to quantify RNA. GAPDH and mRNA primers were synthesized by Shanghai Sangong Company, whereas U6 and miRNA primers were prepared by Guangzhou Ruibo Biotechnology Co., LTD., with specific sequences shown in Table 1.

**Table 1:** Primer sequences used for qRT-PCR in this study.

Gene name	Primer sequence (5′–3′)
miR-122-5p	Forward: TATTCGCACTGGATACGACACAAAC
	Reverse: GCCCGTGGAGTGTGACAATGGT
CS	Forward: GTGACTGGCACCCAATGTTT
	Reverse: GTCCAGAACATCCTCTGCCT
U6	Forward: GCTTCGGCAGCACATATACTAAAAT
	Reverse: CGCTTCACGAATTTGCGTGTCAT
GAPDH	Forward: GAGAAGTATGACAACAGCCTC
	Reverse: ATGGACTG TGGTCATGAGTC

### Western blotting

The RIPA reagent (ApplyGen, China) was used for extracting total cell or tissue protein according to the manufacturer’s instructions. The BCA kit (SolarBio, China) was used for measuring the concentration of extracted protein. Equal amounts of protein samples were loaded onto 10% SDS-PAGE for electrophoresis. The proteins were transferred to PVDF membranes (Beyotime, China) after separation. The protein-loaded membrane was incubated with 5% skim milk for 1 h, and then incubated with the corresponding primary antibody (CS ab129095, Abcam; GPX4 Cat No.67763-1-lg, Proteintech; ACSL4 ab155282, Abcam) overnight in a 4 °C refrigerator. To facilitate incubation and exposure, the protein-loaded membrane was cut into small bands with the corresponding molecular weight and placed in the primary antibody for incubation. After incubation in TBS containing Tween-20, the membranes were incubated with the corresponding secondary antibody (BST17E13B17F54, Boster) for 1 h. Finally, the protein bands were visualized using a chemiluminescence kit (UElandy, China) and analyzed by Image Lab 5.2.1 software (Bio-Rad, USA).

### Cell counting kit-8 (CCK-8) assay

A total of 5 × 10^3^ cells were added into each well of 96-well plates. Then, 10 µl CCK-8 reagent (HanBio, China) was added to each well and incubated at 37 °C for 2 h. Finally, the optical density (OD) at 450 nm was measured using a microplate reader (Thermo, USA).

### Clone formation assay

A total of 500 cells were added into each well of a 6-well plate and cultured for 14 days. The cells were stained with methyl violet solution (SolarBio, China) for 30 min. Colonies containing more than 50 cells were counted under a microscope (Olympus, Japan).

### EDU assay

A total of 1 × 10^4^ cells were added into each well of a 96-well plate. EDU working solution (UElandy, China) was added to each well and incubated at room temperature for 2 h. Cells were fixed with 4% neutral paraformaldehyde after washing with glycine. Permeabilization was performed using 3% BSA, followed by staining with Apollo dye and Hoechst33342. After washing with PBS, images were collected under a fluorescence microscope (Olympus, Japan).

#### Wound healing assay

A total of 2 × 10^5^ cells were added into each well of a 6-well plate. Wounds were created on the surface of cells with a pipette tip after growing to 90% confluence. Wound width was measured at 0 and 48 h using a microscope at 100× magnification. Wound closure degree represents cell migration ability.

#### Transwell assay

Experiments were performed using a Transwell chamber with 8 µm micropores. Cells (2 × 10^4^ cells/well without Matrigel, 5 × 10^4^ cells/well with Matrigel) were added into the upper Transwell chamber (Bio-Rad, USA) containing 200 μl of FBS-free culture medium, with or without Matrigel (Corning, USA). A volume of 600 μl culture medium containing FBS was added to the lower chamber. Non-migratory cells in the upper chamber were removed and stained with methylene violet (SolarBio, China) for 30 min after 24 h. Finally, images were collected under an inverted microscope (Olympus, Japan).

#### Malondialdehyde, Fe^2+^, and glutathione assays

Cells were broken by sonification and the supernatant was collected. A BCA kit (SolarBio, China) was used to measure the protein concentration in the supernatant. The levels of malondialdehyde (MDA) (NjjcBio, China), Fe^2+^ (Leagene, China), and glutathione (GSH) (NjjcBio, China) were then detected using the corresponding commercial kits.

#### ROS measurements

Cells were incubated with 5 μM dihydroethidium (DHE) (Beyotime, China) for 30 min. Then, the cells were incubated with DAPI (Solarbio, China) for 30 min after cleaning with PBS three times. Finally, images were acquired under a fluorescence microscope (Olympus, Japan) after washing with PBS.

#### Transmission electron microscope experiment

Cells were fixed using 2.5% glutaraldehyde fixative for 4 h, followed by 2% osmic acid fixative for 1.5 h. The cells were then placed on ice, dehydrated in an alcohol gradient, and then placed in a mixture of acetone + resin (1:2) for embedding and drying in the dryer. The embedded blocks were cut into 70 nm slices, mounted on a copper net, and dyed twice with 2% uranium acetate and lead citrate. Images were acquired using transmission electron microscopy.

#### Dual luciferase gene reporter assay

Wild-type and mutant CS constructs were obtained from Hanbio Co., LTD (ShangHai). According to the instructions, cells were co-transfected with wt-CS (or mut-CS) and miR-122-5p mimics (or miR-NC) using the R-fect transfection reagent (Biodai, China). After 48 h of transfection, luciferase activity was measured with dual luciferase gene reporter kit (TransGen Biotech, China).

#### Immunofluorescence assay

A total of 2 × 10^4^ cells were seeded into each well of a 24-well plate. Then, cells were fixed with 4% paraformaldehyde for 30 min. Next, 5% Triton X-100 was added and incubated for 20 min at room temperature, followed by blocking in goat serum was added to block for 1 h. The cells were then incubated with primary antibody at 4 °C overnight, followed by incubation with fluorescent secondary antibody in the dark for 1 h. Finally, images were collected under a fluorescence microscope (Olympus, Japan) after washing with PBS.

#### Xenografted mouse model

CNE-2Z cells with miR-122-5p silencing or overexpression (approximately 7 × 10^6^ cells) were mixed with Matrigel and injected into the right axilla of nude mice subcutaneously. After the tumor volume reached 50 mm^3^, 15 mg/kg erastin (MCE, USA) was injected intraperitoneally every other day, and the same dose of solvent was injected intraperitoneally into the control group. Tumor diameter was measured every other day. The nude mice were sacrificed 21 days after cell inoculation or when the tumor volume exceeded 1500 mm^3^. Tumor tissues were excised and partially fixed with 4% paraformaldehyde for subsequent experiments.

#### Caudal venous transfer model

CNE-2Z cells with miR-122-5p silencing or overexpression (approximately 2 × 10^6^ cells) were injected into the tail vein of nude mice. One week after cell inoculation, 15 mg/kg erastin was injected intraperitoneally every other day. Body weight of mice was closely monitored for approximately 4 weeks after cell inoculation. The nude mice were sacrificed when body weight decreased significantly (They lost more than 20% of their normal body weight). Additionally, we used a microscope to count the metastases through multi-person verification, and we confirmed our findings with H&E staining.

#### Immunohistochemistry

The tissue samples were embedded in paraffin wax and cut into 5 μm slices. The sections were incubated in goat serum for 30 min, followed by overnight incubation with primary antibody against CS (1:100, Abcam) at 4 °C overnight. Then, the sections were incubated with immunoglobulin G secondary antibody (1:200; Beyotime, China) for 30 min. Next, the sections were incubated with horseradish peroxidase-labeled streptavidin, and the immune reaction was visualized with diaminobenzidine (Solarbio, China). Hematoxylin staining was performed. Finally, images were collected under a microscope (Olympus, Japan) after cleaning with PBS.

#### Hematoxylin and eosin (H&E) staining

The tissue samples were fixed with 4% paraformaldehyde for 24 h, and dehydrated with a series of increasing concentrations of ethanol. The tissues were then embedded in melted paraffin for 30 min and sectioned at a thickness of 5 μm. The sections were dewaxed with xylene and rehydrated with a series of decreasing concentrations of ethanol. Hematoxylin was used for nuclear staining, while eosin was used for cytoplasmic staining. The sections were dehydrated with absolute ethanol. Finally, images were collected under a microscope (Olympus, Japan).

#### Statistical analysis

Data in the study were presented as the mean ± standard deviation and analyzed using SPSS 26.0 (SPSS Inc., USA) and GraphPad 7.0 (Graphpad Software LLC, USA). Differences between groups were compared using the T-test or analysis of variance. *P* < 0.05 was considered statistically significant.

#### Ethical approval

The tissue samples utilized in this study were obtained with the informed consent of patients or their legal guardians and approved by the Ethics Committee of the Second Affiliated Hospital of Nanchang University. Our work was carried out in conformity with the Declaration of Helsinki. The Ethics Committee of the Institutional Animal Care and Use Committee of Nanchang Royo Biotech Co,. Ltd approved the animal research, which was carried out in conformity with the ARRIVE Guidelines and the Care and Use of Laboratory Animals Guidelines.

## Results

### miR-122-5p regulates lipid metabolism in NPC cells

**Figure 1:**
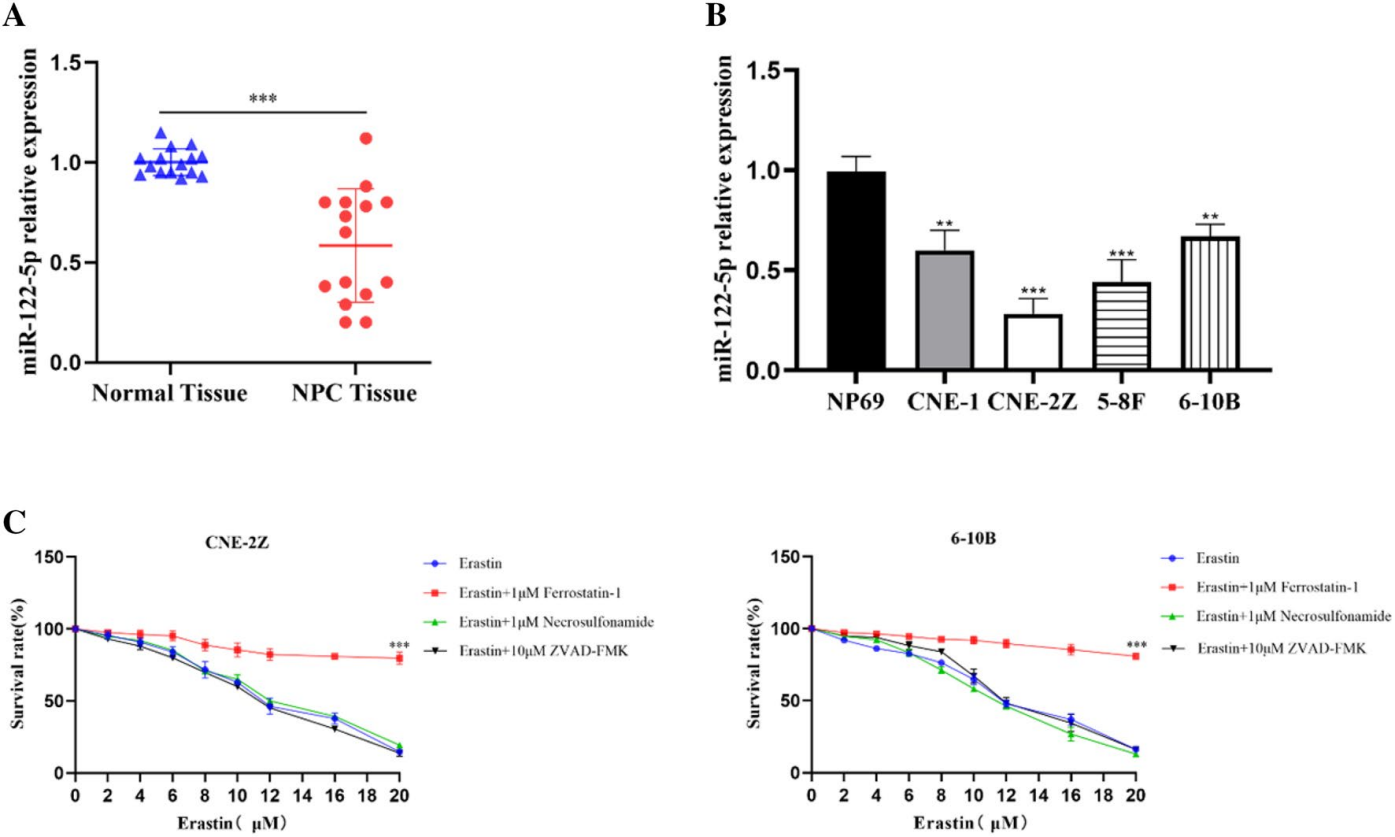
miR-122-5p expression in NPC tissues (**A**) and cells (**B**) was detected by qRT-PCR. The CCK-8 assay showed that erastin-induced ferroptosis in NPC cells (**C**) (All data is repeated 3 times. *:*P* < 0.05; **:*P* < 0.01; ***:*P* < 0.001).

**Figure 2:**
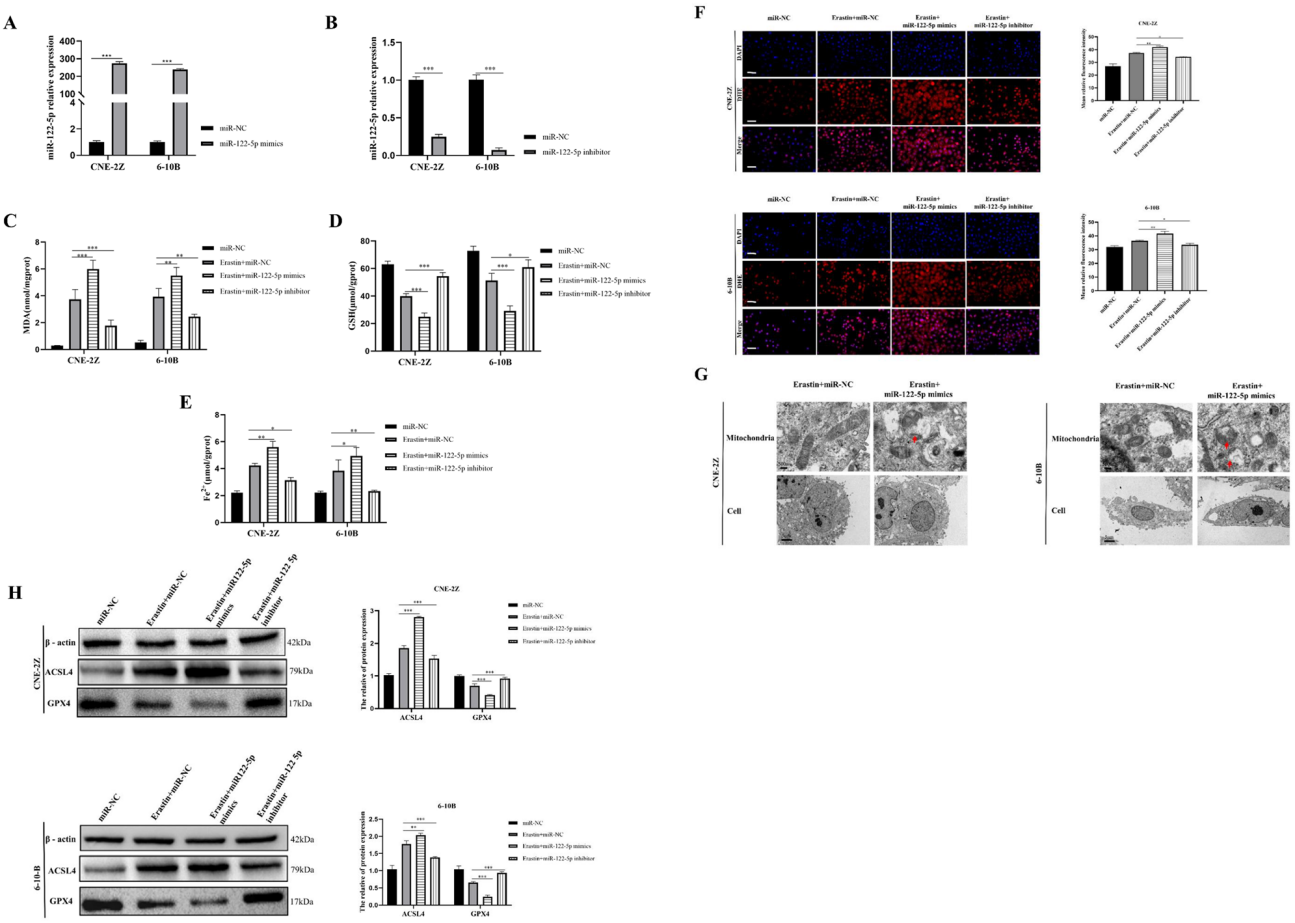
miR-122-5p affected erastin-induced ferroptosis in NPC cells. qRT-PCR showed that cells were effectively transfected with miR-122-5p mimics (**A**) and inhibitor (**B**). The effects of miR-122-5p on MDA (**C**), GSH (**D**), Fe^2+^ (**E**), ROS (**F**), mitochondrial morphology (**G**), and ferroptosis related proteins (**H**) were detected (**D**: The scale is 50 μm; *:*P* < 0.05; **:*P* < 0.01; ***:*P* < 0.001).

The results of qRT-PCR showed that miR-122-5p expression was downregulated in both NPC tissues and cells (Fig. 1A,B). Subsequent experiments were performed using CNE-2Z cells, which have relatively low miR-122-5p expression, and 6-10B cells, which have relatively high miR-122-5p expression among NPC cells. The results of the CCK8 assay showed that earsin induced cell death was reversed by ferrostatin-1, but not by necrosulfonamide (an inhibitor of necroptosis) or ZVAD-FMK (an inhibitor of apoptosis) in NPC cells (Fig. 1C). This indicates that easrin can induce ferroptosis in CNE-2Z and 6-10B cells. miR-122-5p mimics and inhibitor were transfected into CNE-2Z and 6-10B cells, and the efficiency of transfection was detected by qRT-PCR (Fig. 2A,B). All cell groups were treated with 10 μM earsin to induce ferroptosis. Upregulation of miR-122-5p increased the levels of MDA, Fe^2+^, and ROS, and decreased GSH (Fig. 2C–F). Transmission electron microscopy revealed that the mitochondria of cells overexpressing miR-122-5p were more wrinkled and had ruptured membranes, and fewer cristae than control cells (Fig. 2G). miR-122-5p overexpression upregulated the ACSL4 protein and downregulated GPX4 protein expression, whereas miR-122-5p knockdown had the opposite effect (Fig. 2H).

### miR-122-5p suppresses the malignant biologic behavior of erastin‐induced NPC cells

**Figure 3:**
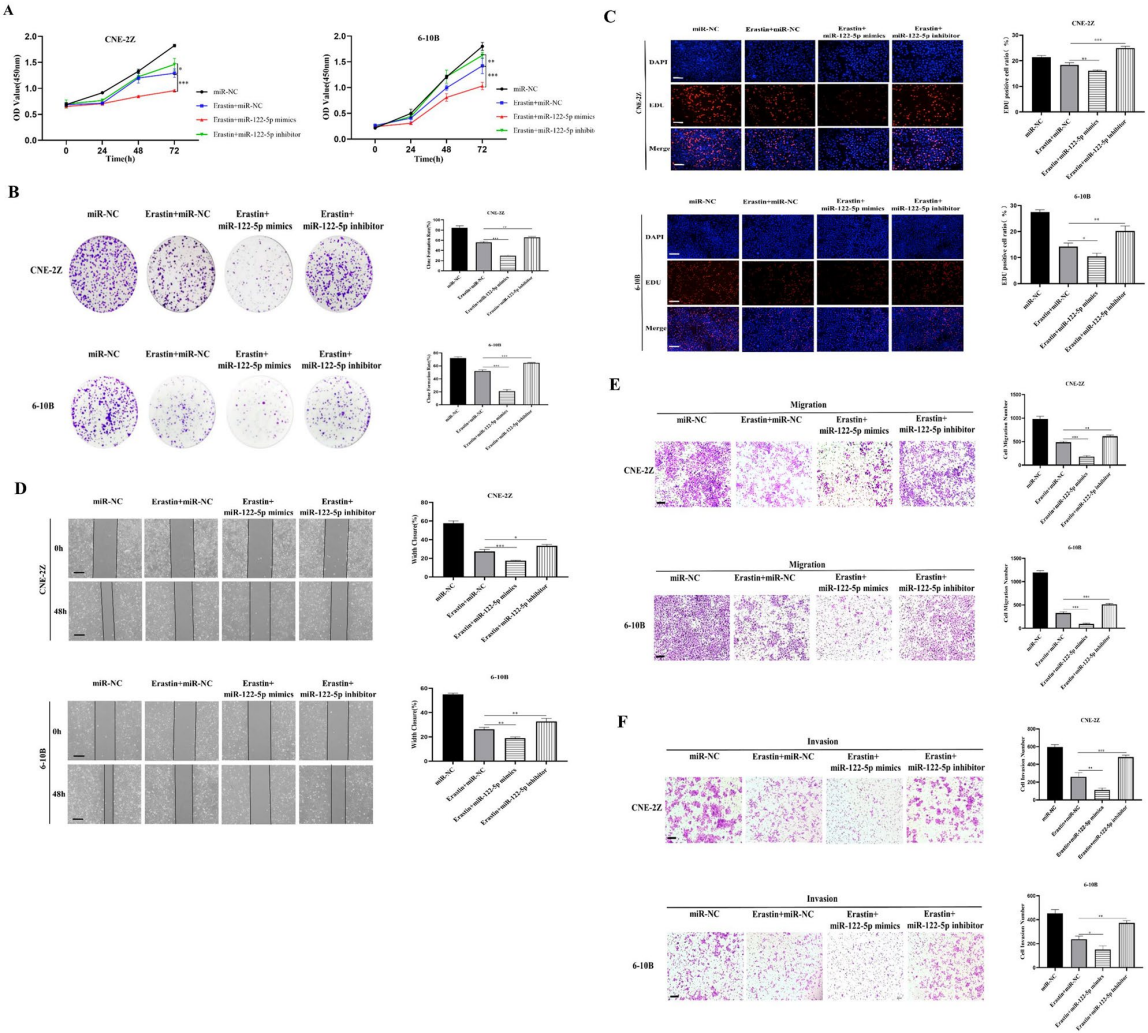
miR-122-5p affects the malignant biologic behavior of erastin-induced NPC cells. The effects of miR-122-5p on cell proliferation, migration, and invasion were examined using the CCK-8 assay (**A**), colony formation assay (**B**), EDU assay (**C**), wound healing assay (**D**), and Transwell assay (**E**,**F**) (**C**–**F**: The scale is 50 μm; *:*P* < 0.05; ** :*P* < 0.01; ***:*P* < 0.001).

**Figure 4:**
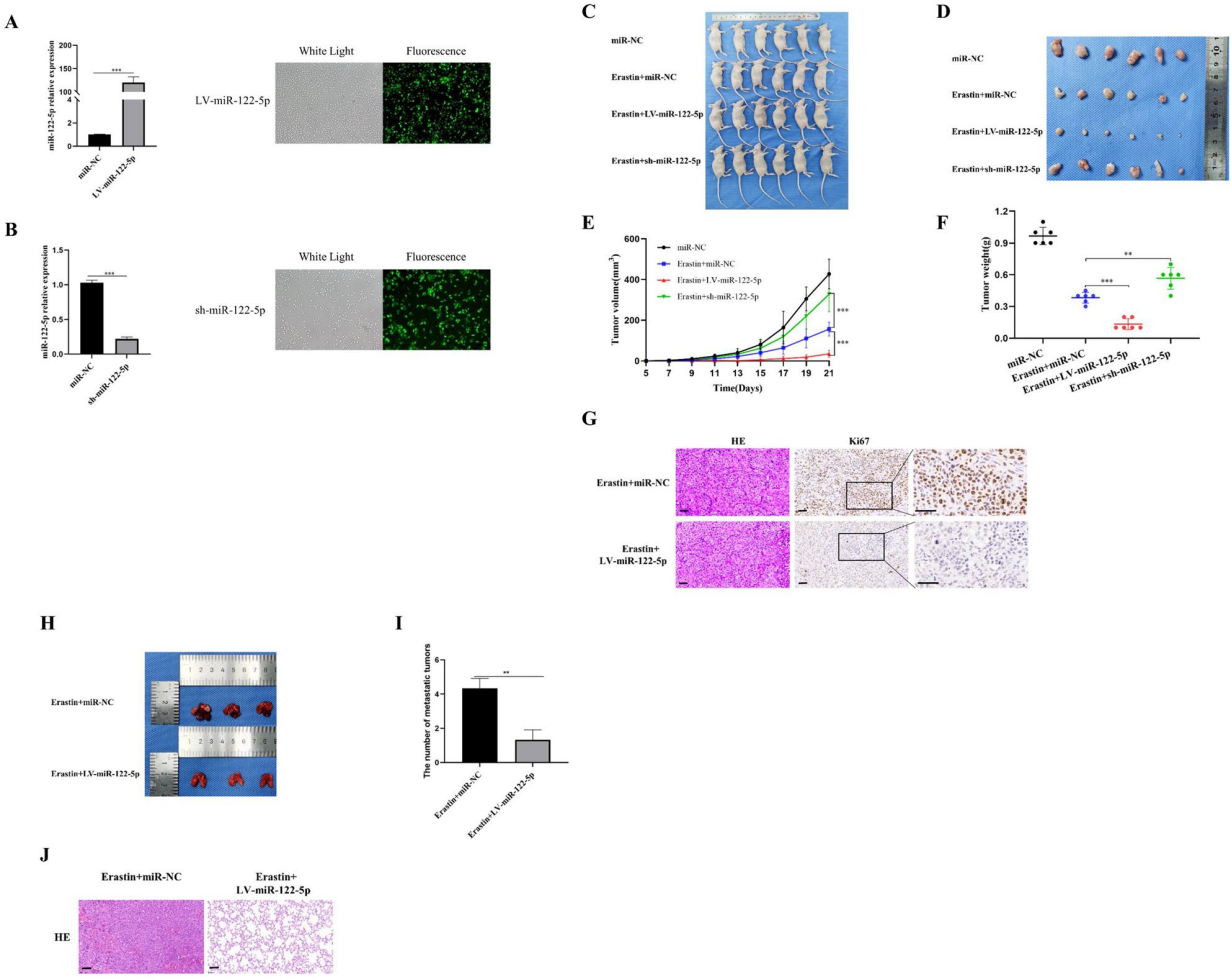
miR-122-5p affects proliferation and metastasis of erastin-inducted NPC cells in vivo. After qRT-PCR confirmed that miR-122-5p was up- or down-regulated in CNE-2Z cells (**A**,**B**). The cells were injected subcutaneously into nude mice. The weight and volume of the tumors were measured after tumor formation (**C**–**F**). Tumor proliferation was detected by immunohistochemical detection of ki67 (**G**). Lung tumor metastasis was detected after caudal intravenous injection of CNE-2Z cells (**H**–**J**) (**G**,**J** : The scale is 50 μm; **:*P* < 0.01; ***:*P* < 0.001). (LV: overexpression; sh: knockdown; NC: control).

The effect of miR-122-5p on the proliferation of erastin-treated CNE-2Z and 6-10B cells was examined using the CCK-8, EDU, and colony formation assays. The results showed that miR-122-5p overexpression significantly reduced cell proliferation, whereas miR-122-5p inhibitors increased cell proliferation (Fig. 3A–C). The wound healing assay and Transwell assay were used to examine the effect of miR-122-5p on migration and invasion erastin-treated NPC cells. The results showed that miR-122-5p overexpression decreased invasion and migration, whereas miR-122-5p inhibition had the opposite effects (Fig. 3D–F). These results suggest that up-regulation of miR-122-5p inhibited the malignant biologic behaviors of NPC cells treated with erastin (such as proliferation, migration, and invasion) As shown in Fig. 4, the inhibitory effect of miR-122-5p overexpression on the proliferation and metastasis of NPC cells was confirmed in erastin-induced nude mice (Fig. 4A–J).

### miR-122-5p directly targets CS in NPC

**Figure 5:**
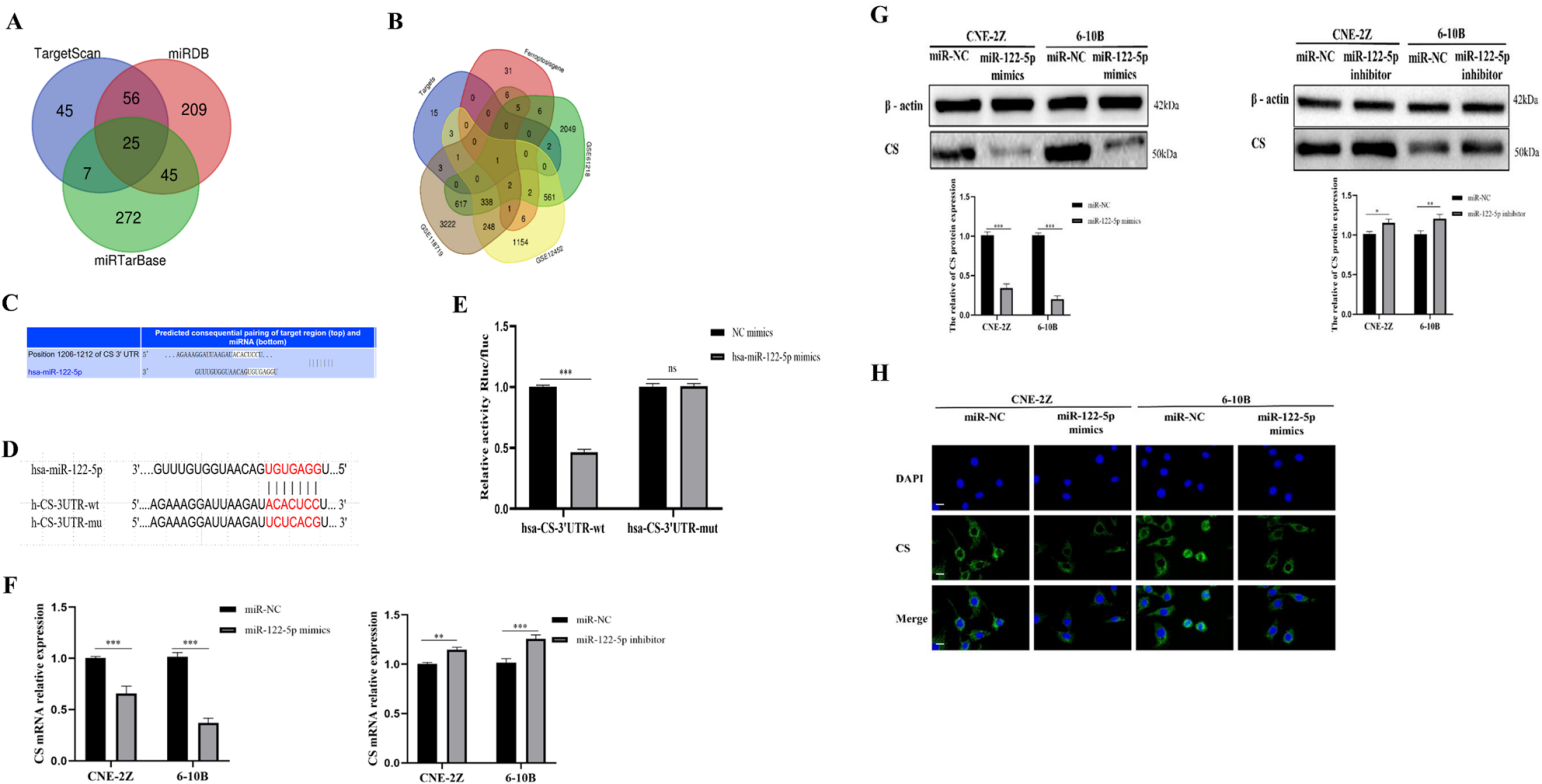
CS is a target of miR-122-5p regulation. Bioinformatics prediction of miR-122-5p target genes (**A**–**C**). The regulation of CS by miR-122-5p was confirmed by dual luciferase reporter gene assay (**D**,**E**), qRT-PCR (**F**), western blotting (**G**), and immunofluorescence (**H**) (**H**: The scale is 50 μm; ns: no significance; *:*P* < 0.05; **:*P* < 0.01; ***:*P* < 0.001).

TargetScan, miRTarBase, and miRDB were used to predict the target genes of miR-122-5p. Then, the target genes were intersected with ferroptosis-related genes^42^ and differentially expressed genes in NPC from the GEO databases (GSE12452, GSE61218, and GSE118719) were used to identify the final target gene, CS (Fig. 5A,B). The binding sites between miR-122-5p and CS were predicted using the TargetScan database. A dual-luciferase reporter gene assay was used to confirm binding between miR-122-5p and CS (Fig. 5C–E). The results of immunofluorescence assay, qRT-PCR, and western blotting confirmed that miR-122-5p negatively regulates CS expression (Fig. 5F–H).

### CS regulates the lipid metabolism of NPC cells

**Figure 6:**
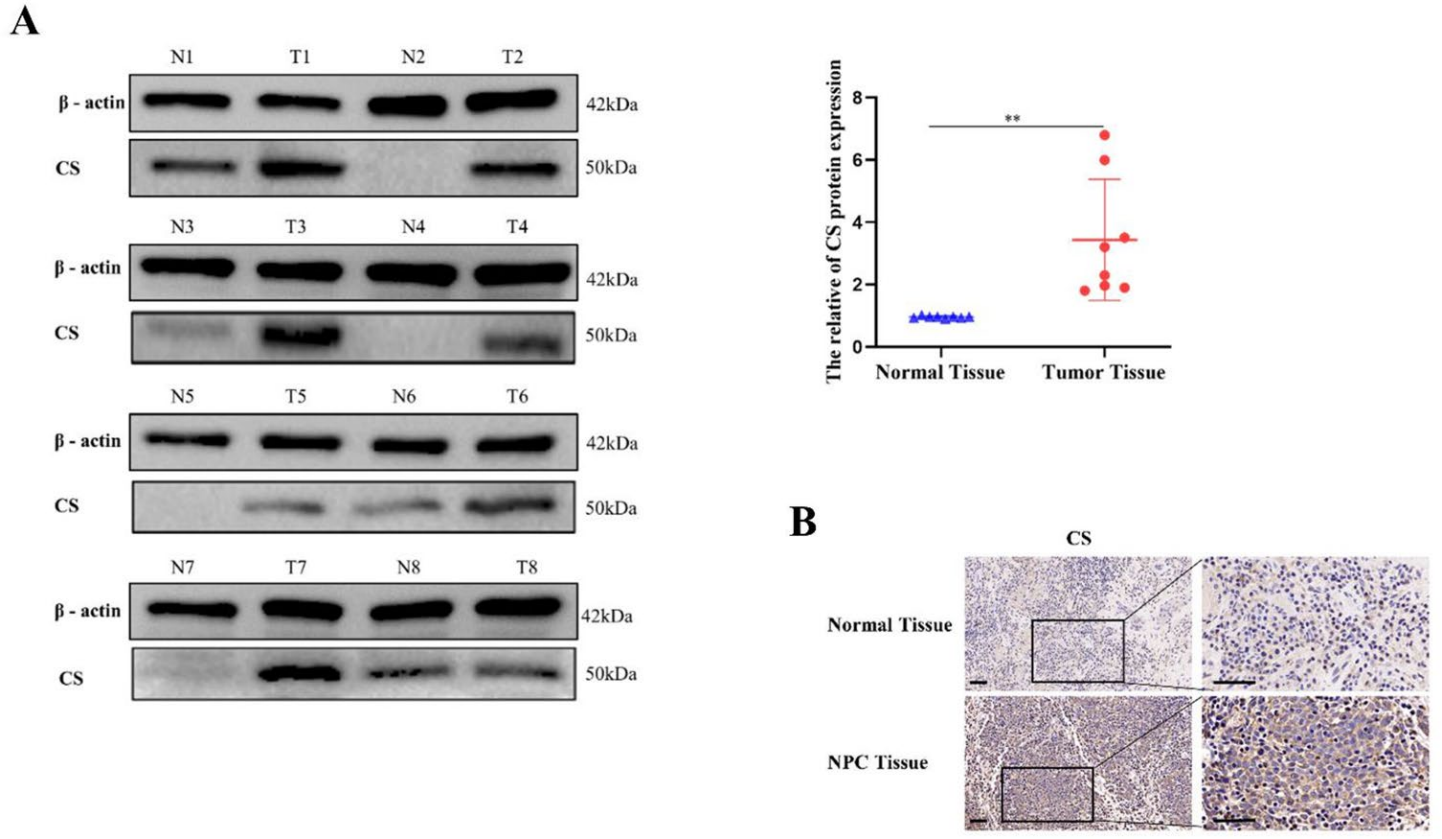
Western blotting (**A**) and immunohistochemistry (**B**) indicated that CS was abundant in NPC tissues (**B**: The scale is 50 μm; **:*P* < 0.01).

**Figure 7:**
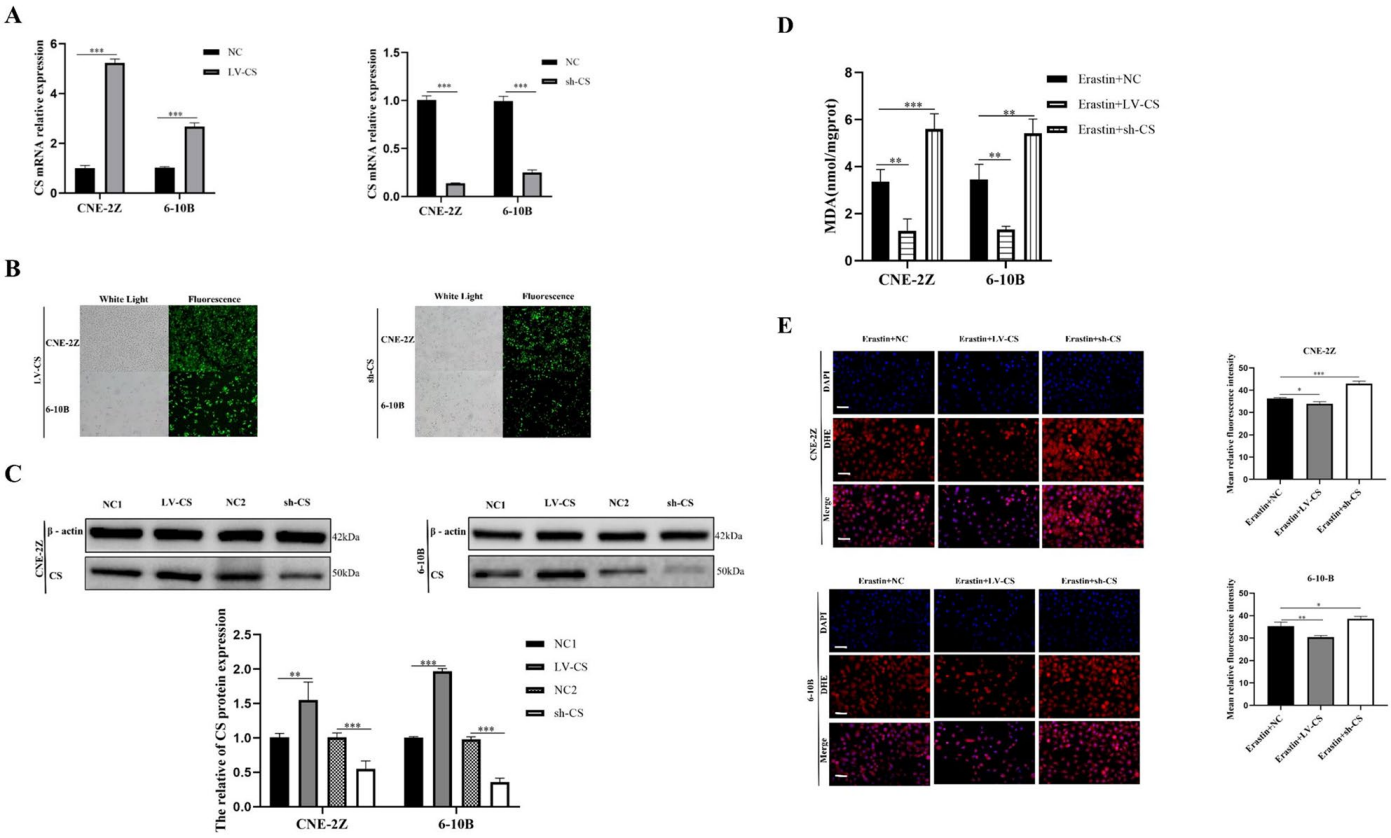
CS decreased erastin-induced ferroptosis in NPC cells. qRT-PCR and western blotting were used to confirm the up-regulation and down-regulation of CS (**A**–**C**). CS affected the levels of MDA (**D**) and ROS (**E**) (**E**: The scale is 50 μm; *:*P* < 0.05; **:*P* < 0.01; ***:*P* < 0.001).

The results of western blotting showed that NPC tissues had higher levels of CS expression than adjacent non-tumor tissues, which was also supported by immunohistochemical assays (Fig. 6A,B). We used lentiviral vectors to overexpress and knock-down CS, and the transfection efficiency was confirmed by qRT-PCR and western blotting (Fig. 7A–C). In NPC cells treated with erastin, CS overexpression decreased the levels of MDA and ROS, suggesting a reduction in ferroptosis. Conversely, the levels of MDA and ROS were higher in cells with down-regulated CS than in control cells (Fig. 7D,E).

### CS promotes the malignant biologic behavior of erastin‐induced NPC cells

**Figure 8:**
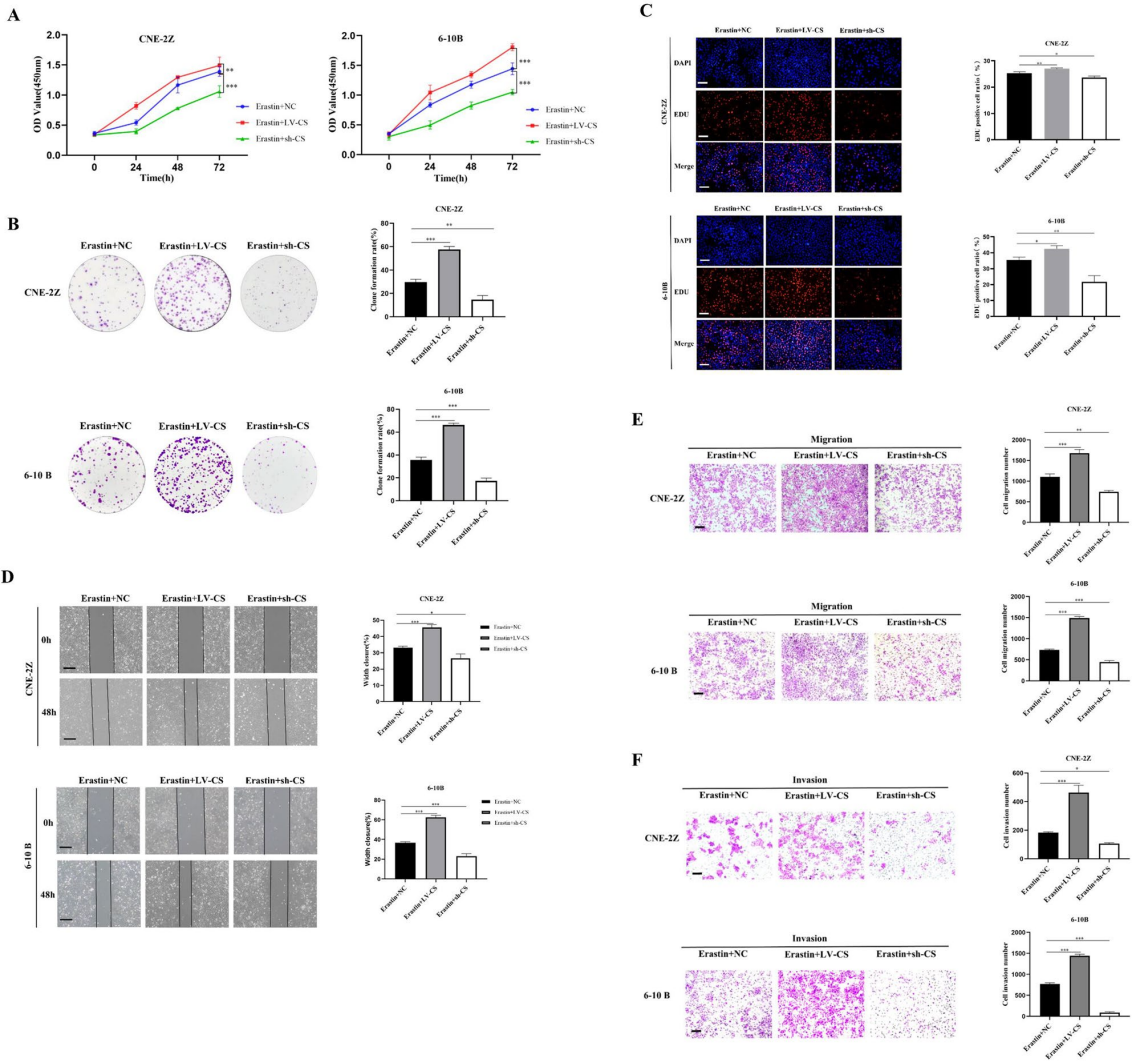
CS promoted the malignant biologic behavior of erastin-induced NPC cells. The effects of CS on cell proliferation, migration, and invasion were detected by the CCK8 assay (**A**), colony formation assay (**B**), EDU assay (**C**), wound healing assay (**D**), and Transwell assay(**E**,**F**) (**C**–**F**:The scale is 50 μm; *:*P* < 0.05; **:*P* < 0.01; ***:*P* < 0.001).

**Figure 9:**
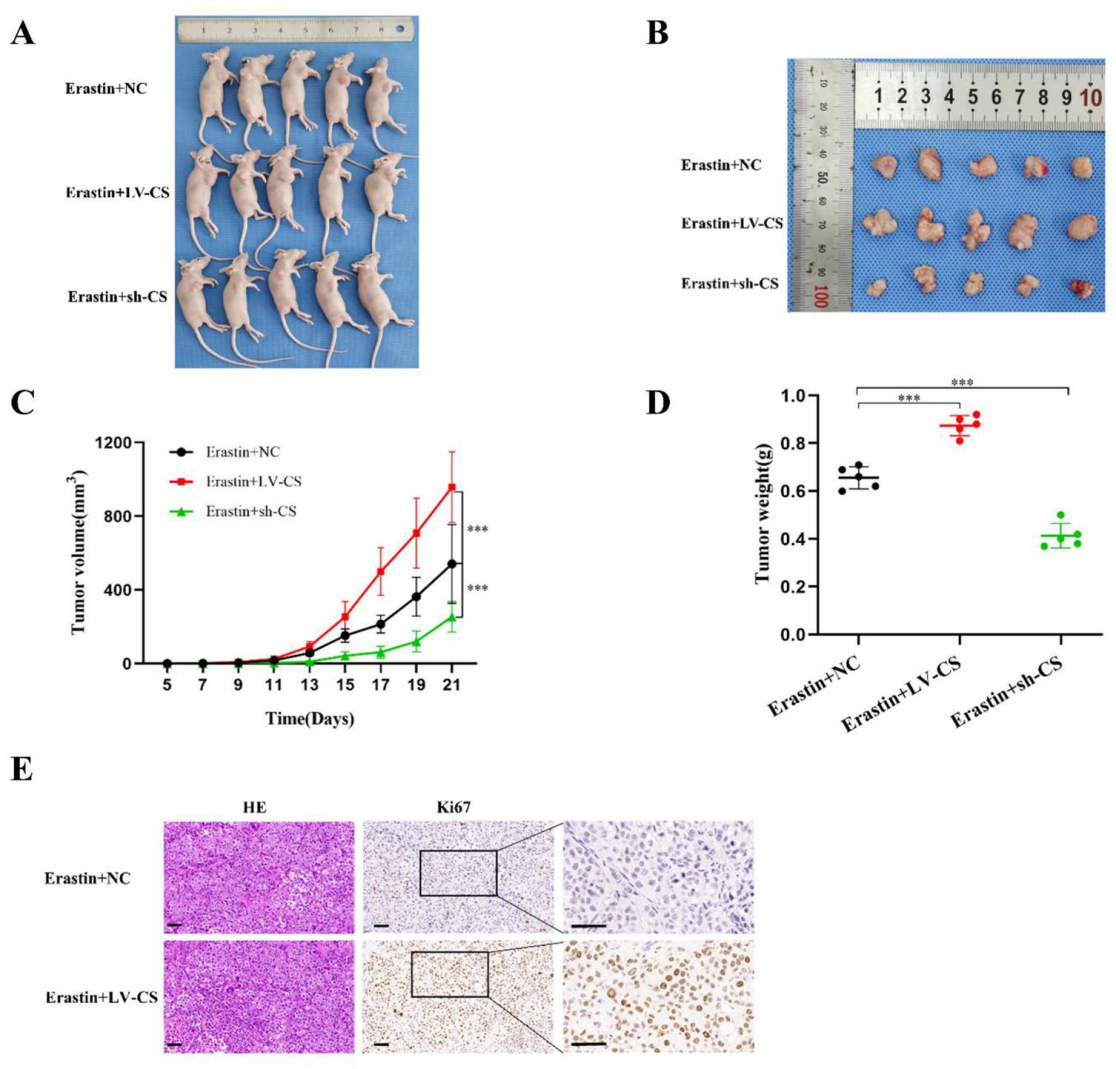
CS stimulated the proliferation of erastin-treated NPC cells in vivo. CNE-2Z cells with CS knockdown or overexpression were injected subcutaneously into nude mice. The weight and volume of tumors were measured after tumor formation (**A**–**D**). Tumor proliferation was detected by immunohistochemi cal detection of ki67 (**E**) (**E**: The scale is 50 μm; ***:*P* < 0.001).

The results of the CCK-8, colony formation, and EDU assays showed that knockdown of CS decreased proliferation, whereas overexpression of CS increased proliferation in erastin-induced NPC cells (Fig. 8A–C). To confirm the effect of CS overexpression on migration and invasion in erastin-induced NPC cells, we performed wound healing and Transwell assays (Fig. 8D–F). As shown in Fig. 9, the role of CS in promoting NPC proliferation was confirmed in an erastin-induced ferroptosis model in nude mice (Fig. 9A–E).

### miR-122-5p promotes ferroptosis through CS

**Figure 10:**
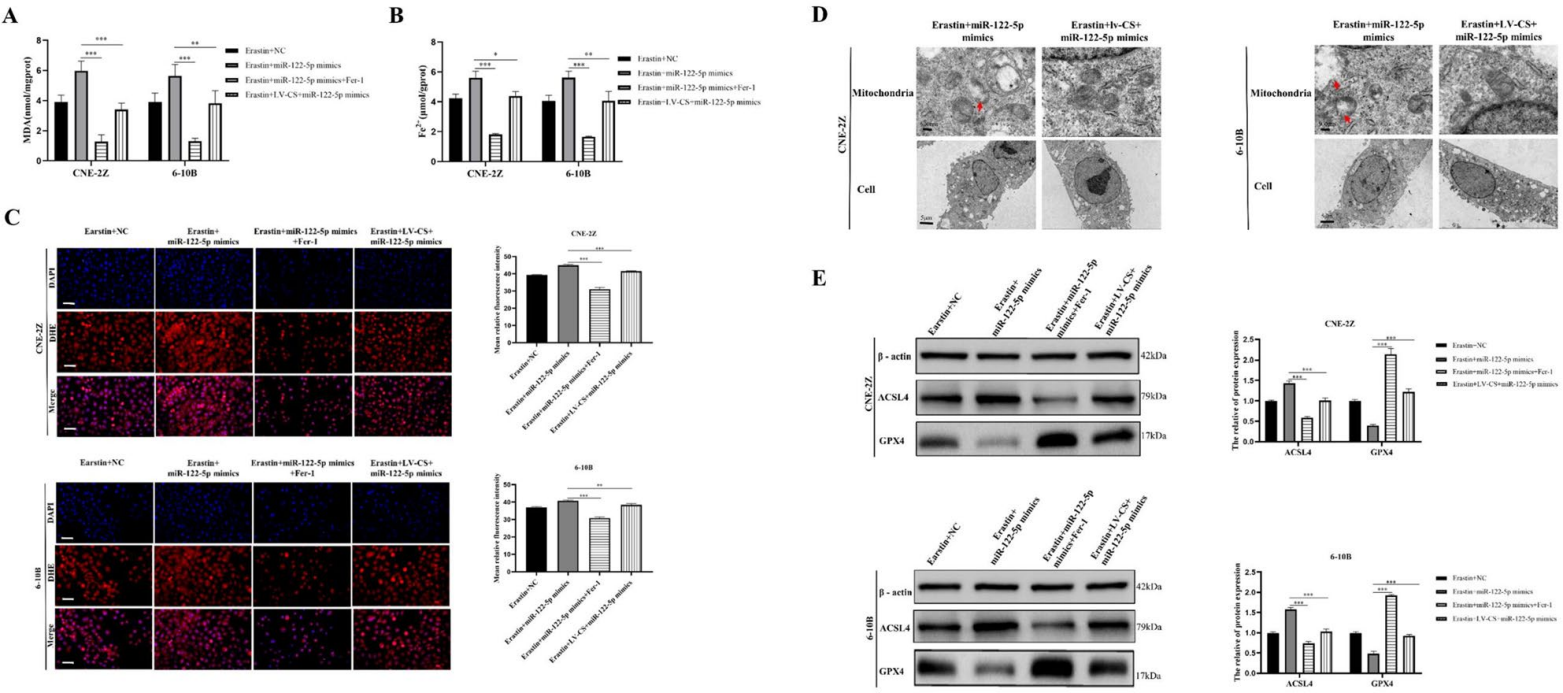
The effect of miR-122-5p on ferroptosis was reduced by Fer-1 and overexpressed CS. Fer-1 and overexpressed CS were found to have effects on MDA (**A**), Fe^2+^ (**B**), ROS (**C**), mitochondrial morphology (**D**), and ferroptosis associated proteins (**E**) (**C**:The scale is 50 μm; *:*P* < 0.05; **:*P* < 0.01; ***:*P* < 0.001).

**Figure 11:**
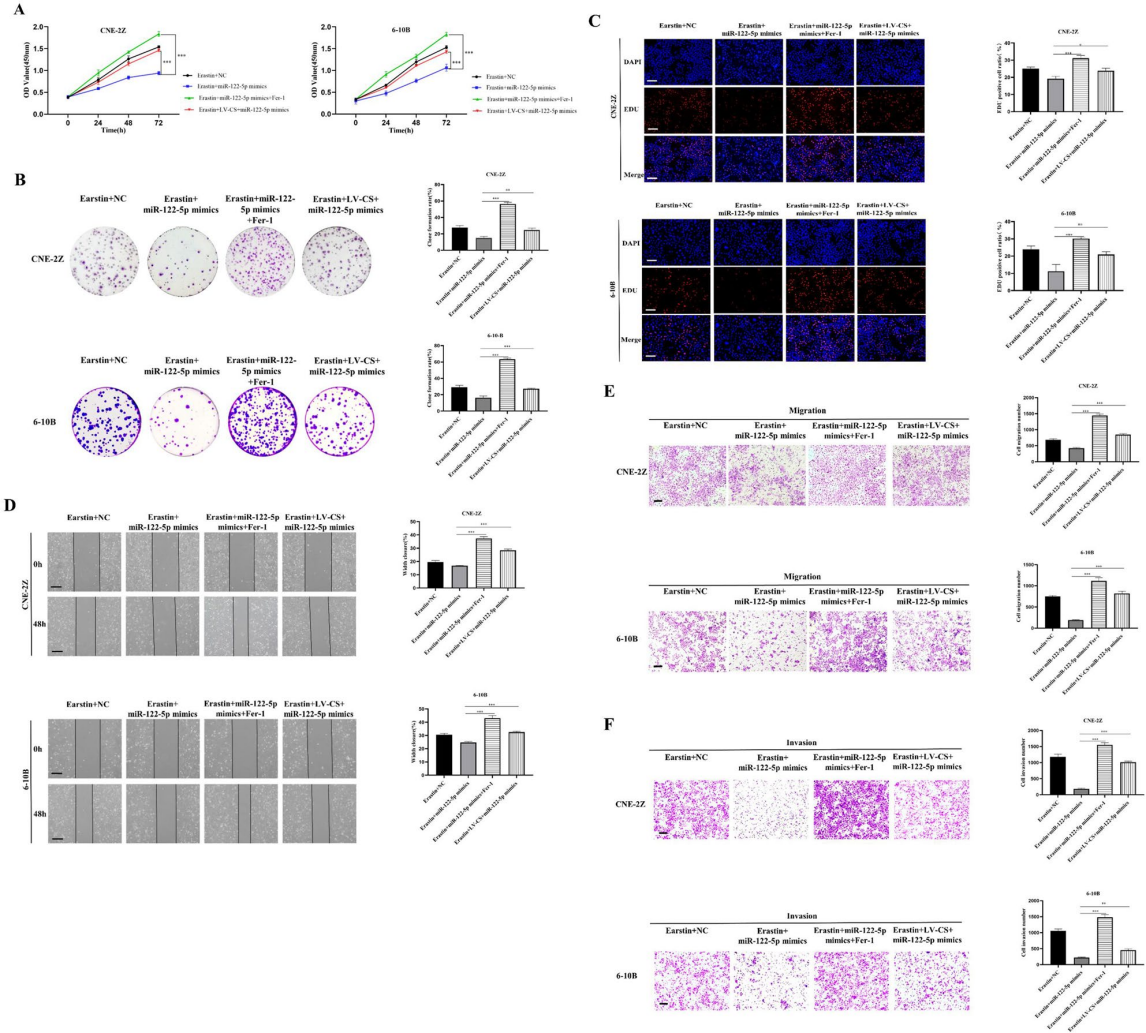
The effects of miR-122-5p on malignant biologic behavior were reduced by Fer-1 and overexpressed CS. The CCK-8 assay (**A**), colony formation assay (**B**), EDU assay (**C**), wound healing assay (**D**), and Transwell assay (**E**,**F**) were used to examine the effects of Fer-1 and overexpressed CS on cell proliferation, migration, and invasion (**C**–**F**: The scale is 50 μm; *:*P* < 0.05; **:*P* < 0.01; ***:*P* < 0.001).

Subsequent experiments were performed using cells treated with 1 μM Fer-1 or with CS overexpression. In the presence of erastin, MDA, Fe^2+^, and ROS levels were lower in cells treated with Fer-1 or overexpressing CS than in cells overexpressing miR-122-5p alone (Fig. 10A–C). Electron microscopy revealed that mitochondrial membrane rupture and shrinkage were restored in cells overexpressing both miR-122-5p and CS (Fig. 10D). Western blotting results also showed that inhibition of ferroptosis or overexpression of CS decreased ACSL4 protein expression and increased GPX4 protein expression (Fig. 10E). The results of the CCK-8, EDU, colony formation, wound healing, and Transwell assays showed that the malignant biologic behavior of cells overexpressing miR-122-5p was enhanced after treatment with Fer-1 or overexpression of CS (Fig. 11A–F).

## Discussion

In this study, we showed that miR-122-5p inhibits the proliferation, migration, and invasion of NPC by promoting erastin-induced ferroptosis. miR-122-5p can target and regulate CS. Upregulation of CS attenuated the effect of miR-122-5p on promoting ferroptosis-as well as the inhibition of NPC proliferation. These results suggest that miR-122-5p functions by targeting CS.

microRNAs can regulate the development of malignant tumors through ferroptosis. For example, Bao et al. found that miR-670-3p inhibits ferroptosis to promote the growth of glioblastoma by targeting ACSL4^43^. Deng et al. showed that miR-324-3p enhances ferroptosis in cisplatin-resistant lung adenocarcinoma A549 cells by targeting GPX4, thereby reversing its resistance^44^. Liu et al. showed that miR-15a-3p targets GPX4 to promote ferroptosis in colorectal cancer^45^.

The inhibitory effect of miR-122-5p on the malignant biologic behavior of NPC has been demonstrated. However, the relationship between miR-122-5p and ferroptosis remains unclear. We hypothesized that miR-122-5p could regulate the malignant biologic behavior of NPC through ferroptosis. The experimental results showed that, up-regulation of miR-122-5p in erastin-induced NPC cells led to an increase in MDA and ROS levels, an increase in Fe^2+^, and a decrease in GSH, suggesting the promotion of ferroptosis. Mitochondrial changes observed under electron microscopy were consistent with ferroptosis promotion. The results of the CCK-8, EDU, colony formation, wound healing, and Transwell assays confirmed that the malignant biologic behavior of erastin-induced NPC cells was significantly inhibited after overexpression of miR-122-5p.

We then investigated the pathway through which miR-122-5p affects NPC through ferroptosis. The TargetScan, miRTarBase, and miRDB databases were used to identify miR-122-5p target genes. The intersecting genes were cross-referenced with ferroptosis-related genes and differentially expressed genes in NPC from the GEO database, and CS was finally identified as the target gene. The targeted regulation between miR-122-5p and CS was verified by dual-luciferase reporter gene assay, qRT-PCR, western-blotting, and immunofluorescence assay.

CS is a dimer composed of monomers containing 20 α-helices and one β-sheet^46^. The CS gene is located on human chromosome 12 and encodes citrate synthase, which catalyzes the synthesis of citrate from acetyl-CoA and oxaloacetic acid. This is an important metabolic reaction, which is the first step in the tricarboxylic acid cycle. CS is involved in energy metabolism, lipid metabolism, glucose metabolism and other important physiological processes in the human body^47,48^. CS is involved in glucose and lipid metabolism, and is closely associated with exercise-related diseases, diabetes, and other metabolic disorders^49,50^. There is evidence that CS affects the development of a variety of malignancies^51,52^. CS and citrate are associated with iron metabolism and lipid peroxidation^53,54^. Increased CS expression was observed in residual Burkitt lymphoma cells derived from doxorubicin and vincristine exposure^55^. A mouse ovarian surface epithelial cancer model showed increased CS activity correlated with cancer progression^56^. Studies in SKOV3 and A2780 cells (ovarian cancer cell lines) indicated that knockdown of the CS gene inhibited cell proliferation, migration, and invasion, and enhanced chemotherapy sensitivity^40^. However, the effect of CS in NPC has been rarely reported.

The results of in vitro and in vivo experiments suggested that upregulation of CS inhibited erastin-induced lipid peroxidation, thereby promoting NPC cell malignant biologic behavior. In 2012, the concept of ferroptosis was proposed by Stockwell, and is characterized by Fe^2+^ dependence, lipid peroxidation, and depletion of REDOX systems. Ferroptosis is involved in various diseases, such as malignant tumors, neurodegeneration, and ischemia–reperfusion injury^57^. As a result, molecules and drugs targeting ferroptosis have become a hot topic of research and development^58^. Current types of ferroptosis inhibitors include lipophilic antioxidants (ferrostatin-1 and liproxstatin-1), iron chelators (deferiprone), and fat-soluble antioxidants (vitamin E). Ferrostatin-1 is a synthetic selective ferroptosis inhibitor that reduces lipid damage to the cell membrane^59^. In addition to overexpressing miR-122-5p, we included groups treated with the ferroptosis inhibitor Fer-1 or overexpressing CS. Compared with the erastin + miR-122-5p mimics group, the levels of MDA, Fe^2+^, and ROS fluorescence intensity were significantly decreased in the erastin + miR-122-5p mimics + Fer-1 group. This indicates that ferrostatin-1 alleviated lipid peroxidation and ferroptosis in NPC. At the same time, the malignant biological behavior of NPC was significantly enhanced, suggesting that the inhibition of NPC was decreased after Fer-1 treatment. In summary, we suggest that miR-122-5p targets CS to inhibit NPC cell proliferation, migration, and invasion through ferroptosis.

## Conclusion

This study showed that erastin specifically induces ferroptosis in NPC cells. miR-122-5p specifically targets CS to promote erastin-induced ferroptosis in NPC cells, thereby inhibiting-malignant biologic behaviors, such as proliferation, migration, and invasion. The miR-122-5p/CS axis of ferroptosis may be an effective target for improving the prognosis of NPC.

### Supplementary Information

Supplementary Figures.

## Data Availability

All data generated or analyzed in this study are included in this article. All data are original results obtained in this study. We did not use previously published data. However, differentially expressed NPC genes from the GEO database (GSE12452/GSE61218/GSE118719) were used to determine the final target gene CS in Fig. 5.
