# Supplementary material for: MiR-122-5p regulates erastin-induced ferroptosis via CS in nasopharyngeal carcinoma
Source: Sci Rep. 2024 May 1;14:10019. doi: 10.1038/s41598-024-59080-w (PMC11063070; doi:10.1038/s41598-024-59080-w)
Supplement: Supplementary file 1 — Supplementary Figures. [file 41598_2024_59080_MOESM1_ESM.pptx]

## Slide 1
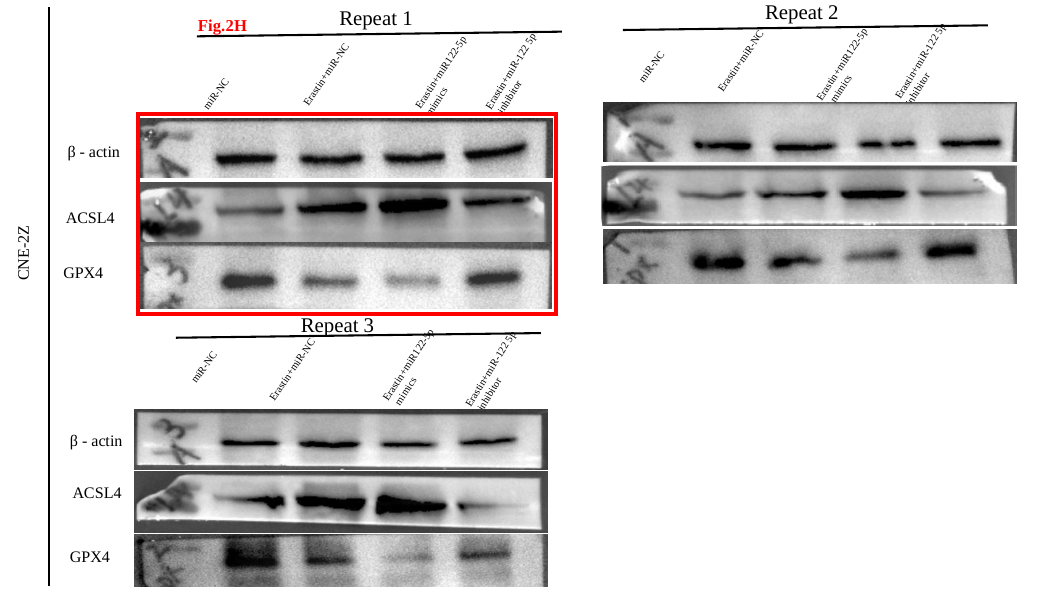

Repeat 2
Repeat 1
Fig.2H
Erastin+miR122-5p
 mimics
Erastin+miR-122 5p
inhibitor
Erastin+miR-122 5p
 inhibitor
miR-NC
Erastin+miR-NC
Erastin+miR122-5p
mimics
miR-NC
Erastin+miR-NC
β - actin
ACSL4
CNE-2Z
GPX4
Repeat 3
Erastin+miR122-5p
 mimics
Erastin+miR-122 5p
 inhibitor
miR-NC
Erastin+miR-NC
β - actin
ACSL4
GPX4

## Slide 2
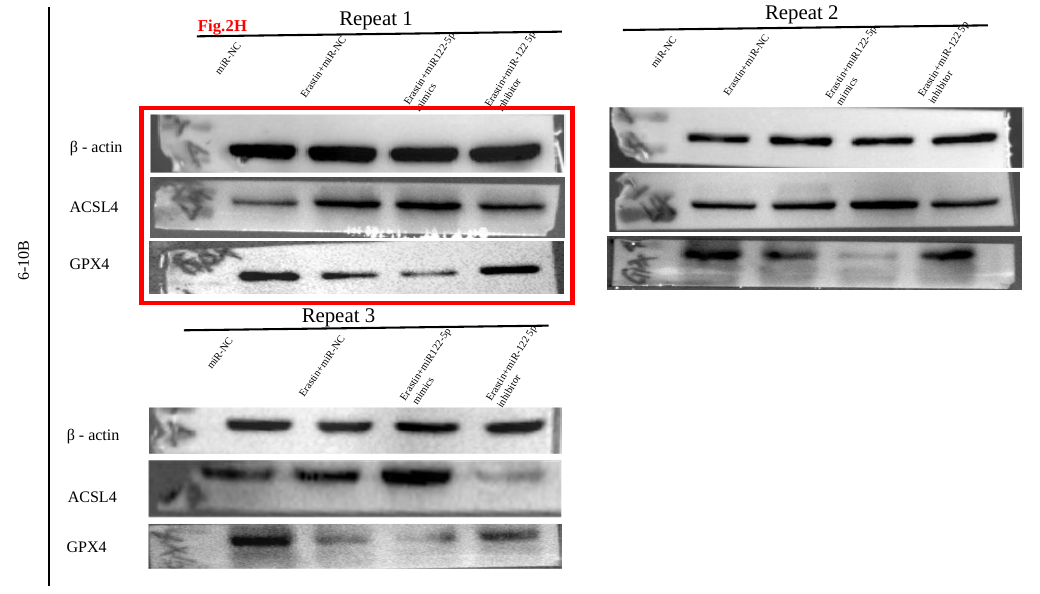

Repeat 2
Repeat 1
Fig.2H
miR-NC
Erastin+miR-122 5p
inhibitor
Erastin+miR122-5p
mimics
miR-NC
Erastin+miR122-5p
mimics
Erastin+miR-122 5p
 inhibitor
Erastin+miR-NC
Erastin+miR-NC
β - actin
ACSL4
6-10B
GPX4
Repeat 3
miR-NC
Erastin+miR122-5p
 mimics
Erastin+miR-122 5p
inhibitor
Erastin+miR-NC
β - actin
ACSL4
GPX4

## Slide 3
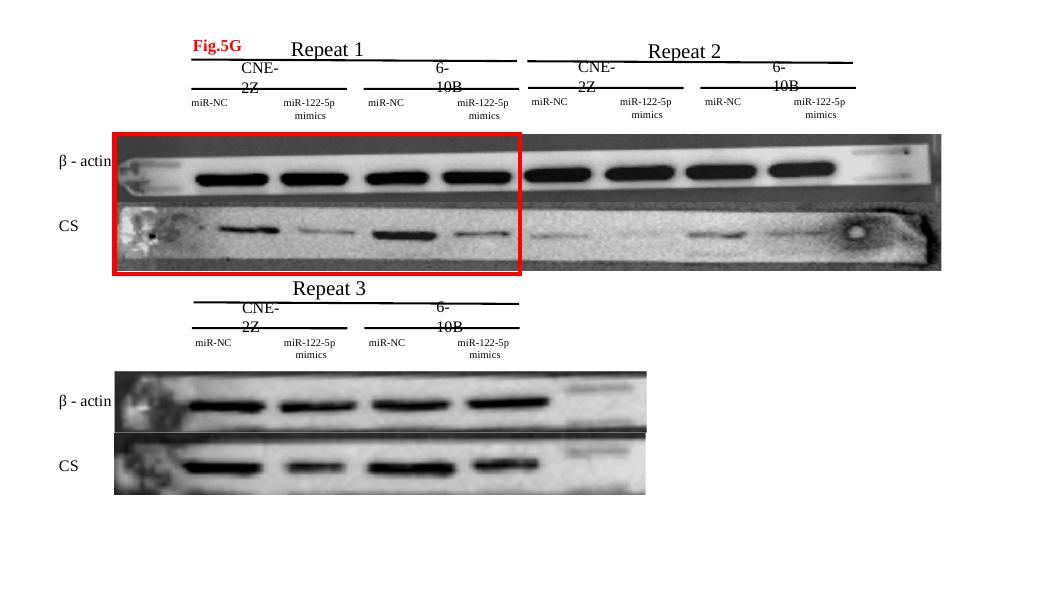

Fig.5G
Repeat 1
Repeat 2
CNE-2Z
CNE-2Z
6-10B
6-10B
miR-NC
miR-NC
miR-122-5p
mimics
miR-122-5p
mimics
miR-NC
miR-NC
miR-122-5p
mimics
miR-122-5p
mimics
β - actin
CS
Repeat 3
CNE-2Z
6-10B
miR-NC
miR-NC
miR-122-5p
mimics
miR-122-5p
mimics
β - actin
CS

## Slide 4
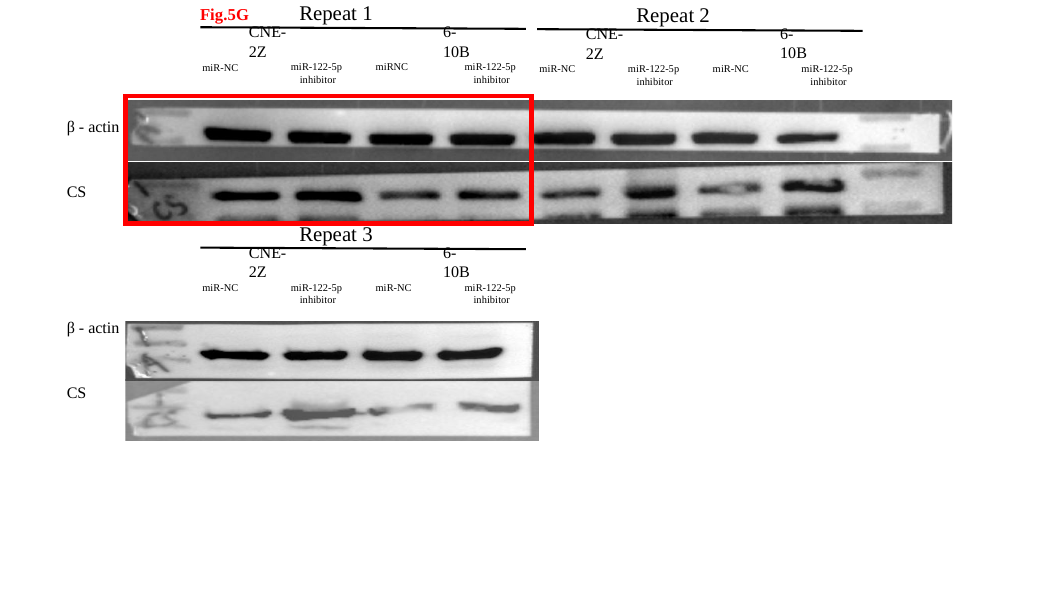

Repeat 1
Repeat 2
Fig.5G
CNE-2Z
CNE-2Z
6-10B
6-10B
miRNC
miR-NC
miR-122-5p
inhibitor
miR-122-5p
inhibitor
miR-NC
miR-NC
miR-122-5p
inhibitor
miR-122-5p
inhibitor
β - actin
CS
Repeat 3
CNE-2Z
6-10B
miR-NC
miR-NC
miR-122-5p
inhibitor
miR-122-5p
inhibitor
β - actin
CS

## Slide 5
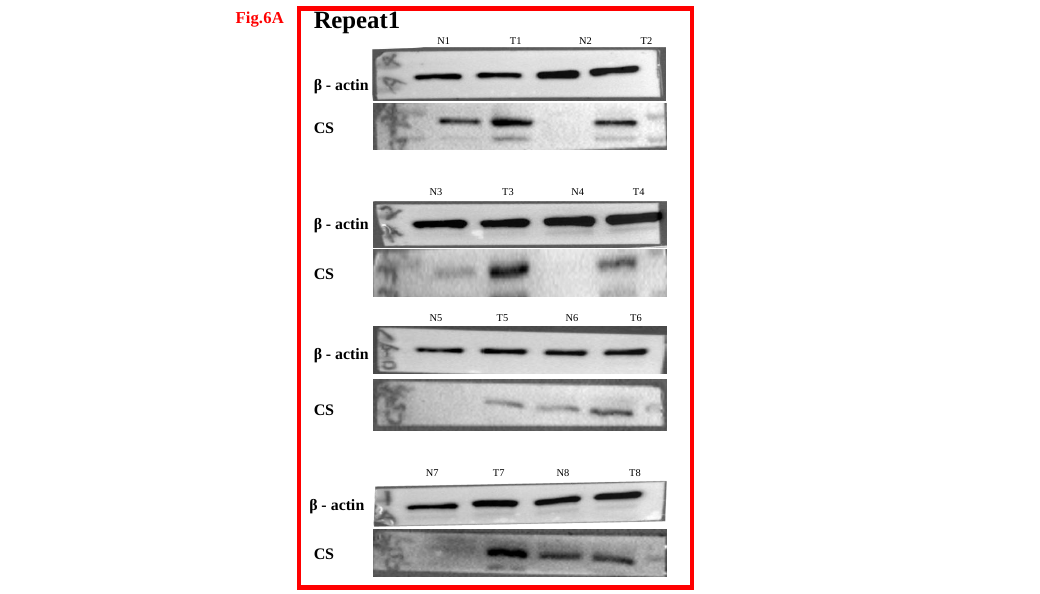

Fig.6A
Repeat1
 N1 T1 N2 T2
β - actin
CS
 N3 T3 N4 T4
β - actin
CS
 N5 T5 N6 T6
β - actin
CS
 N7 T7 N8 T8
β - actin
CS

## Slide 6
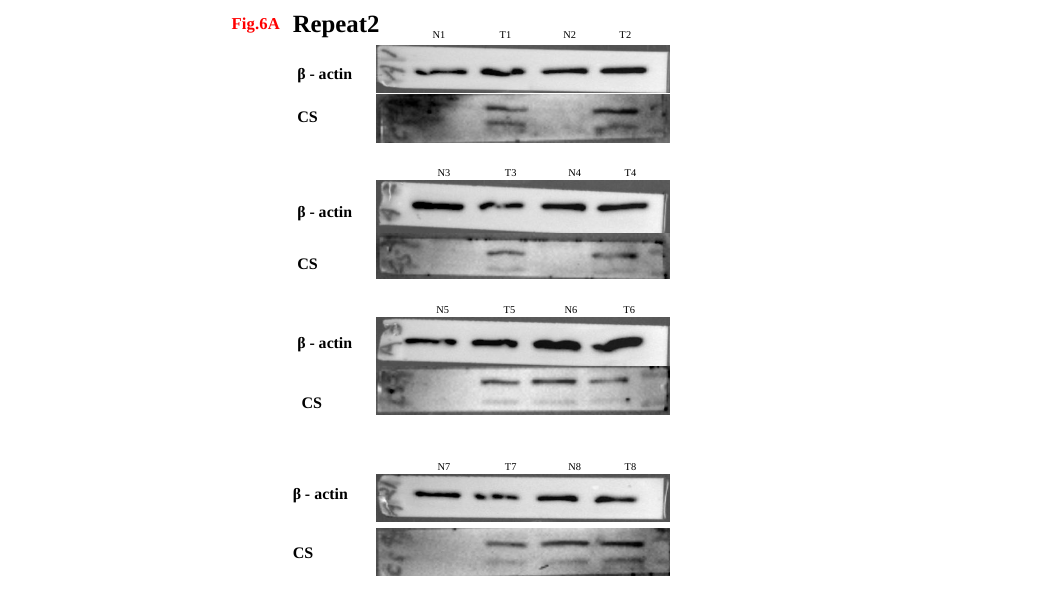

Repeat2
 N1 T1 N2 T2
β - actin
CS
 N3 T3 N4 T4
β - actin
CS
 N5 T5 N6 T6
β - actin
CS
 N7 T7 N8 T8
β - actin
CS
Fig.6A

## Slide 7
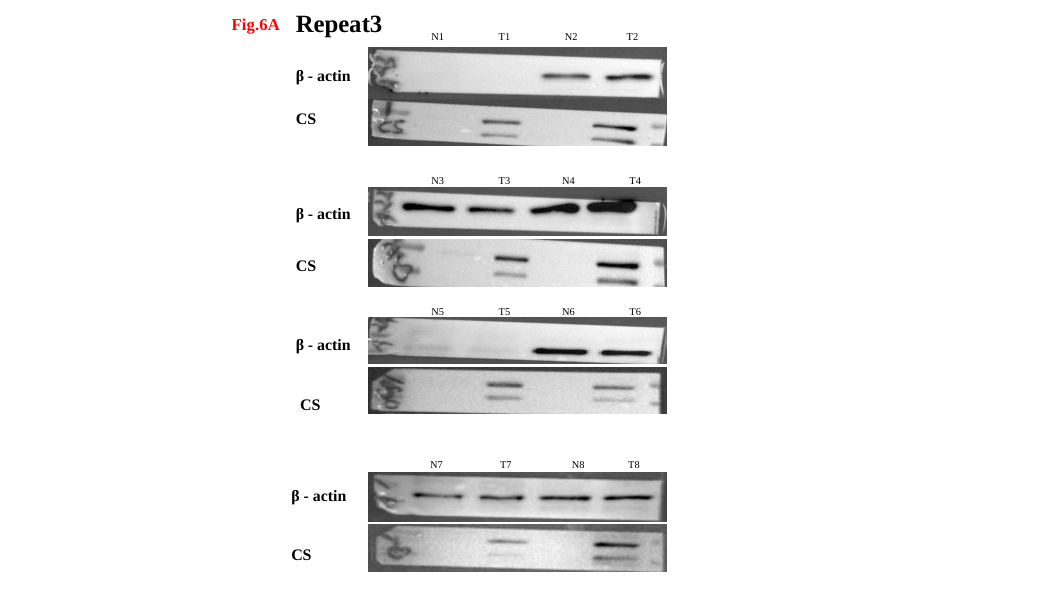

Repeat3
 N1 T1 N2 T2
β - actin
CS
 N3 T3 N4 T4
β - actin
CS
 N5 T5 N6 T6
β - actin
CS
 N7 T7 N8 T8
β - actin
CS
Fig.6A

## Slide 8
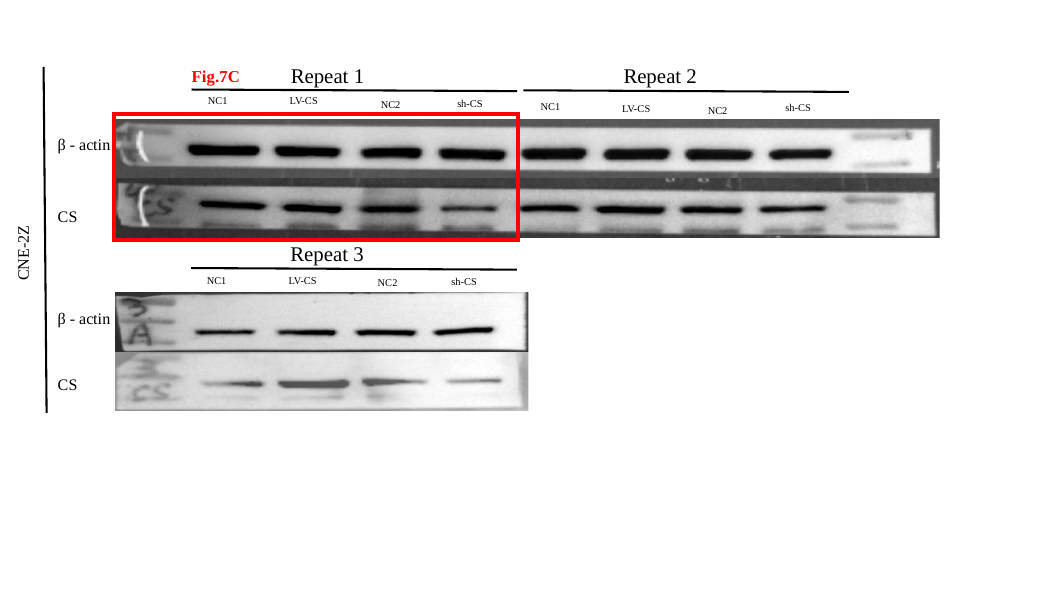

Repeat 1
Repeat 2
Fig.7C
NC1
LV-CS
sh-CS
NC2
NC1
sh-CS
LV-CS
NC2
β - actin
CS
CNE-2Z
Repeat 3
NC1
LV-CS
sh-CS
NC2
β - actin
CS

## Slide 9
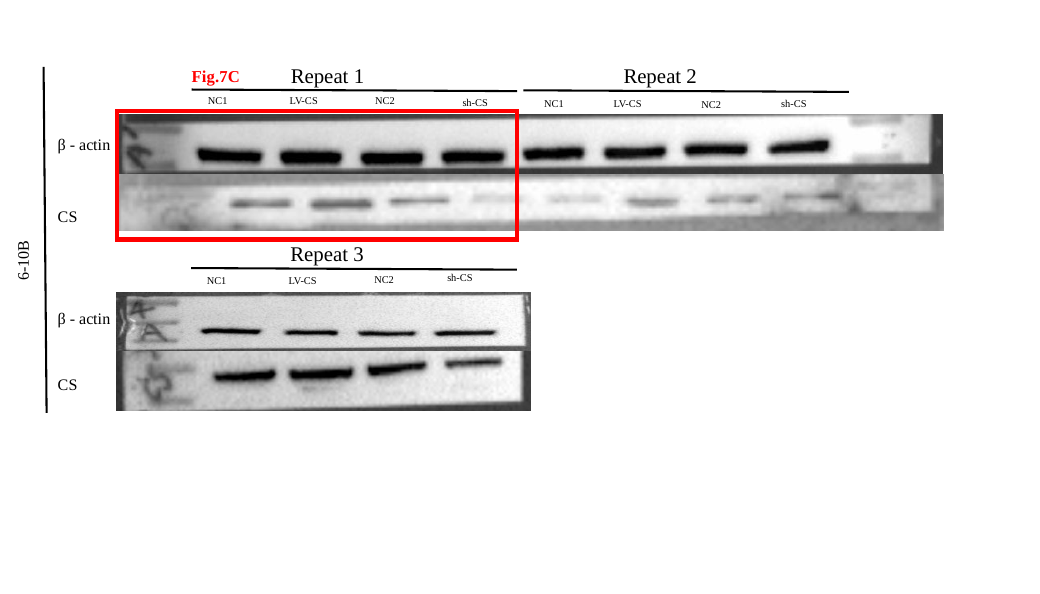

Repeat 1
Repeat 2
Fig.7C
NC2
NC1
LV-CS
sh-CS
LV-CS
NC1
sh-CS
NC2
β - actin
CS
6-10B
Repeat 3
sh-CS
NC2
NC1
LV-CS
β - actin
CS

## Slide 10
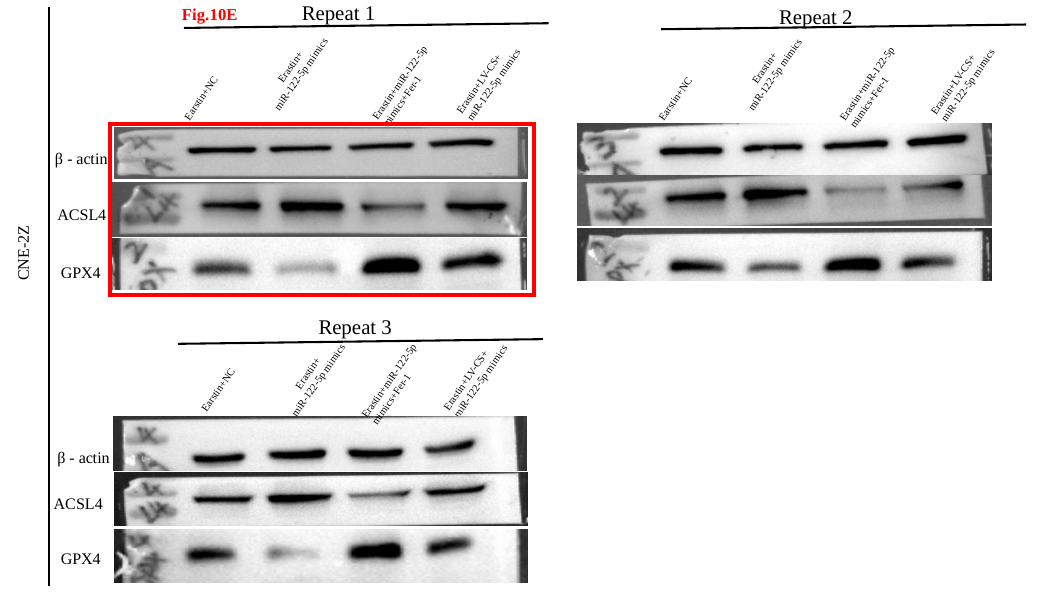

Repeat 1
Repeat 2
Fig.10E
Erastin+LV-CS+
miR-122-5p mimics
Erastin+LV-CS+
miR-122-5p mimics
Erastin+
miR-122-5p mimics
Erastin+
miR-122-5p mimics
Erastin+miR-122-5p mimics+Fer-1
Erastin+miR-122-5p mimics+Fer-1
Earstin+NC
Earstin+NC
β - actin
ACSL4
CNE-2Z
GPX4
Repeat 3
Erastin+LV-CS+
miR-122-5p mimics
Erastin+miR-122-5p mimics+Fer-1
Erastin+
miR-122-5p mimics
Earstin+NC
β - actin
ACSL4
GPX4

## Slide 11
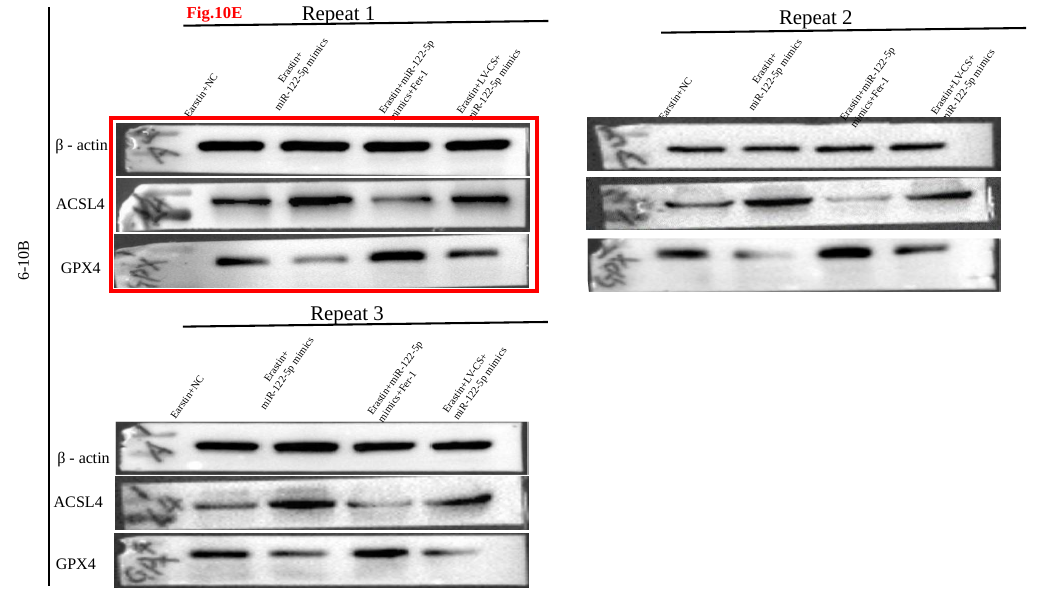

Repeat 1
Fig.10E
Repeat 2
Erastin+LV-CS+
miR-122-5p mimics
Erastin+LV-CS+
miR-122-5p mimics
Erastin+miR-122-5p mimics+Fer-1
Erastin+
miR-122-5p mimics
Erastin+
miR-122-5p mimics
Erastin+miR-122-5p mimics+Fer-1
Earstin+NC
Earstin+NC
β - actin
ACSL4
6-10B
GPX4
Repeat 3
Erastin+LV-CS+
miR-122-5p mimics
Erastin+
miR-122-5p mimics
Erastin+miR-122-5p mimics+Fer-1
Earstin+NC
β - actin
ACSL4
GPX4
